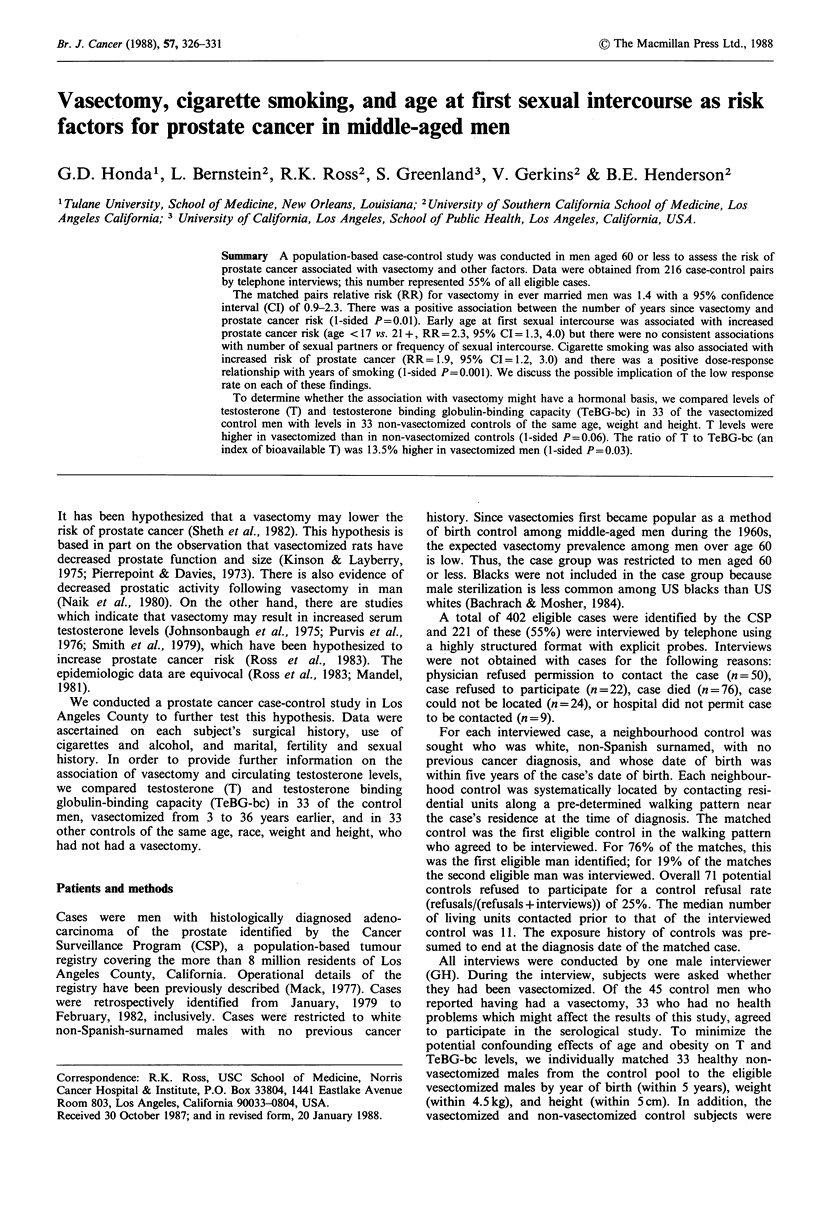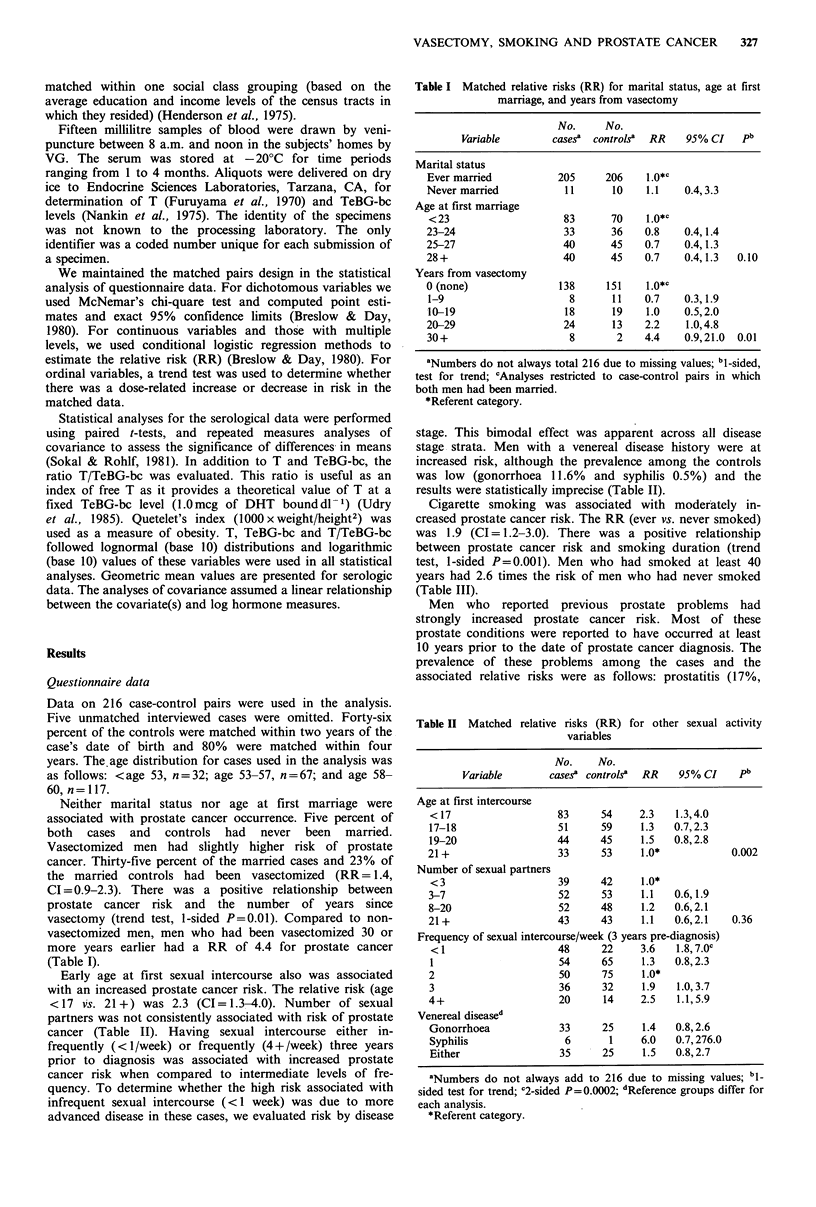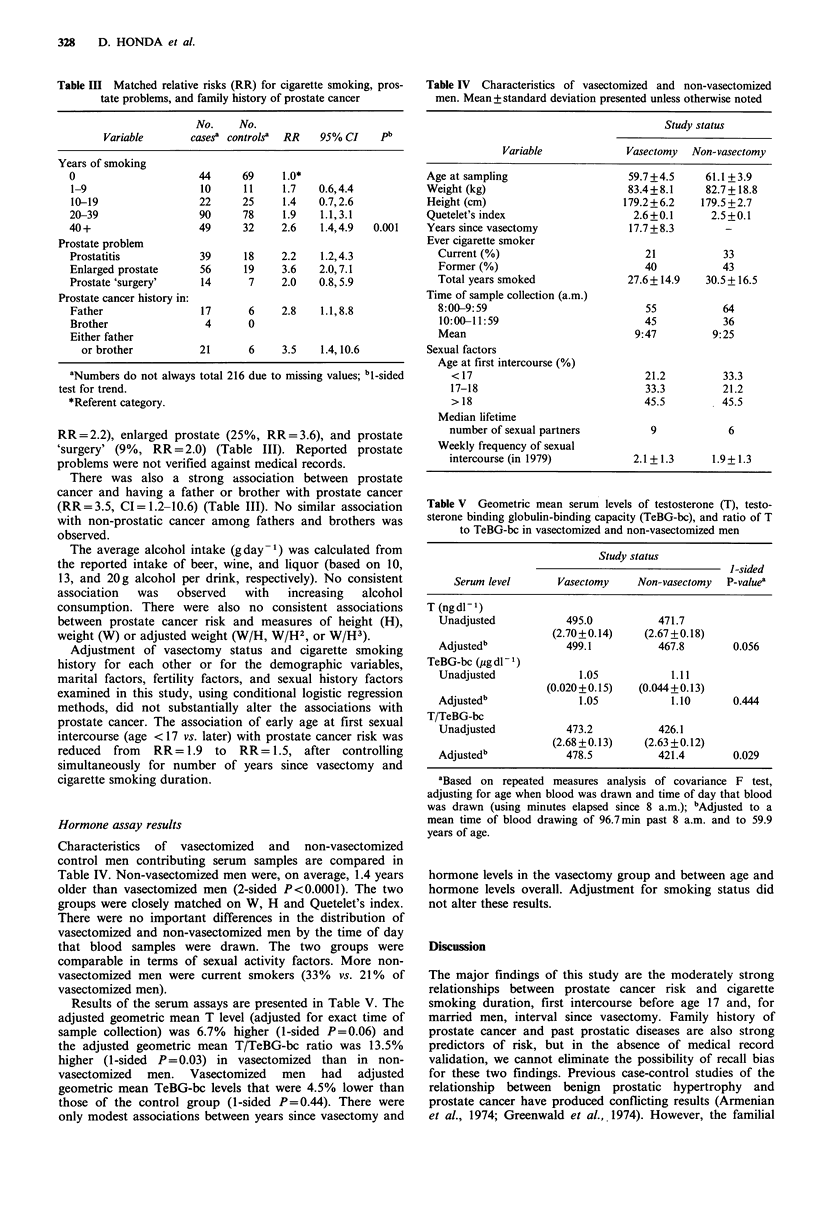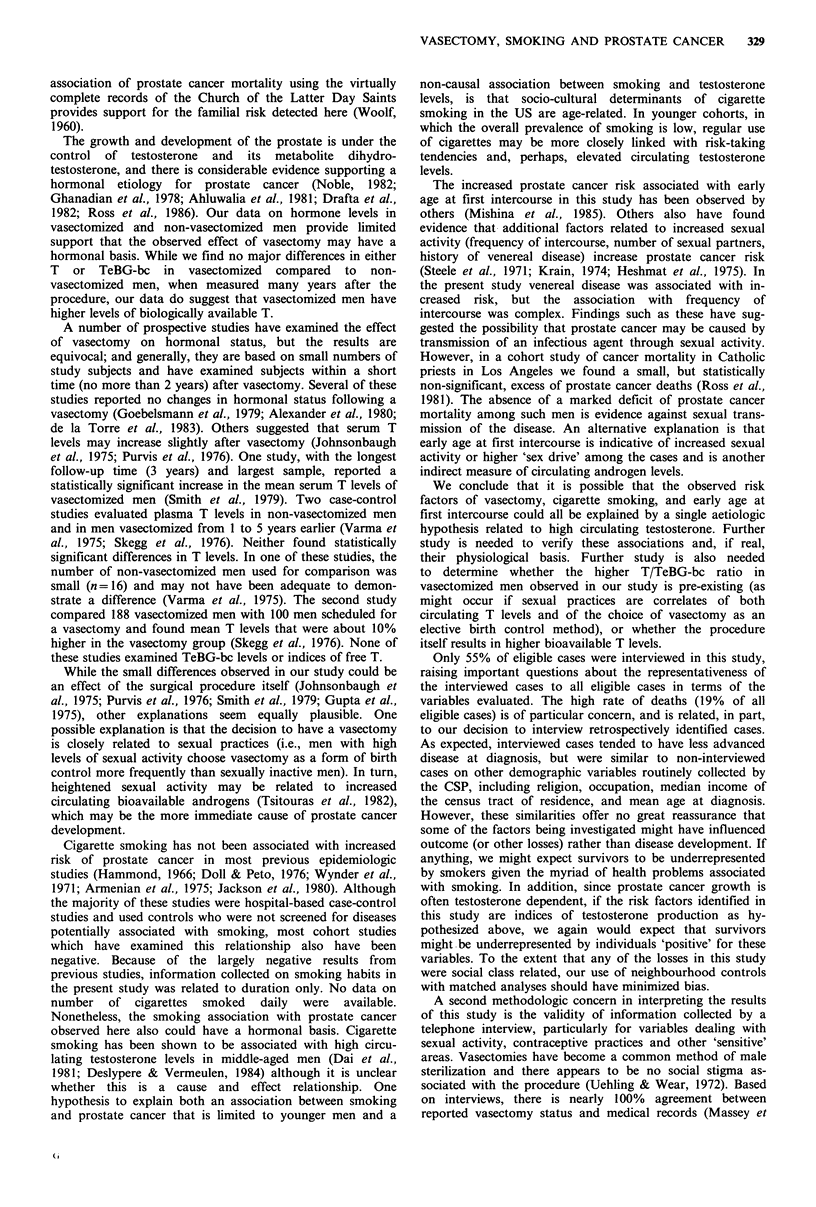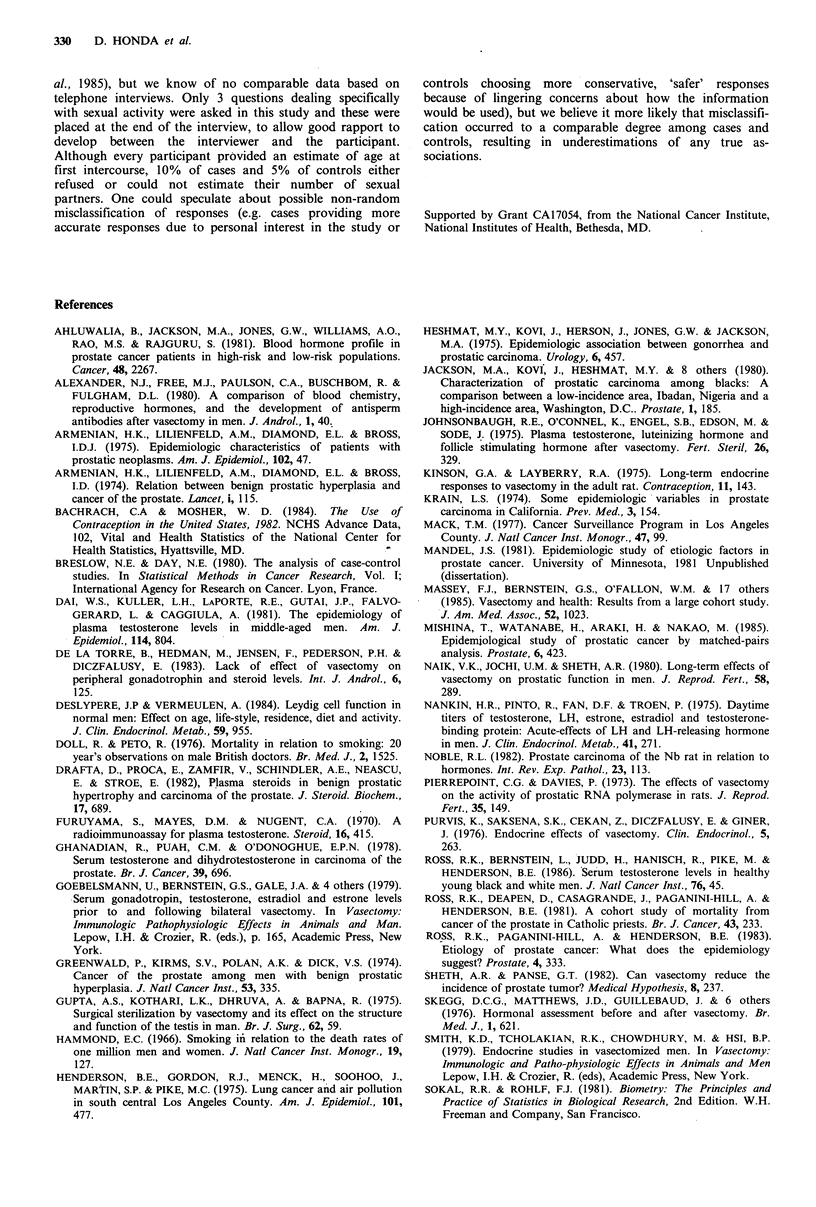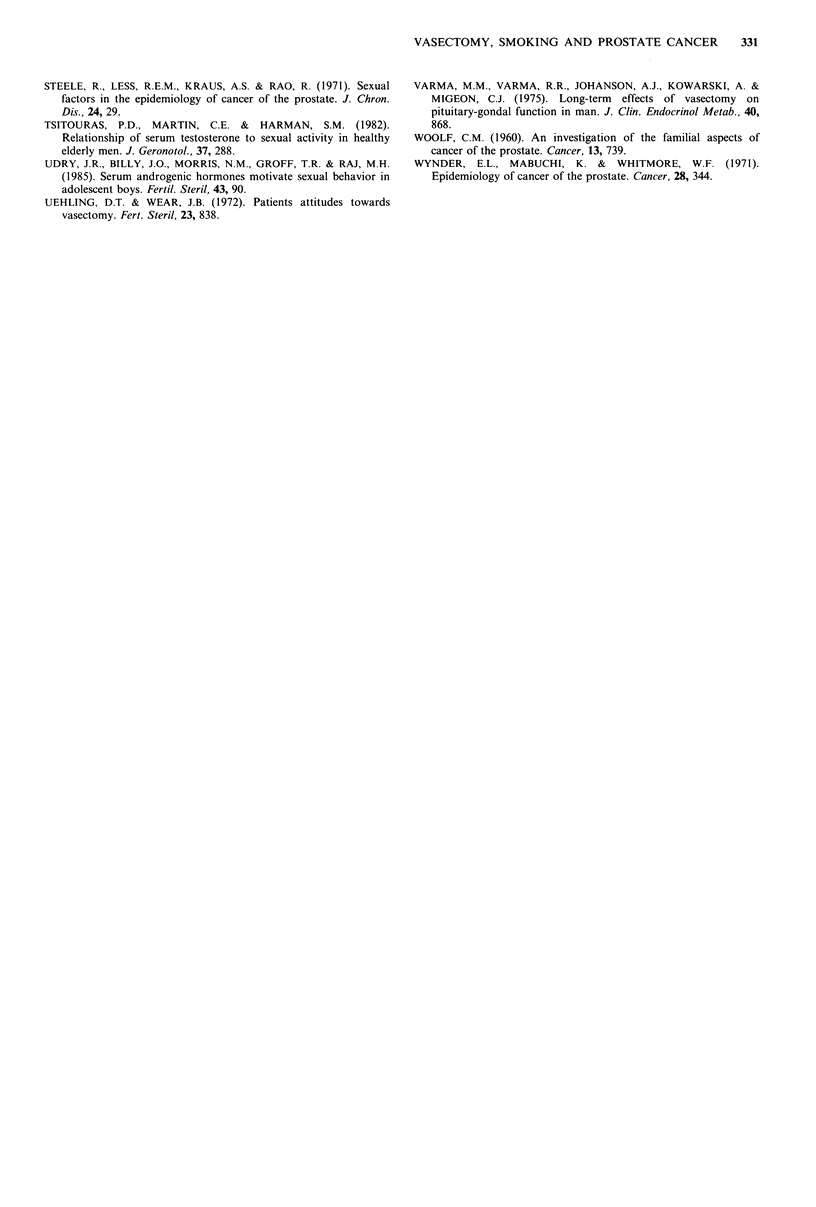# Vasectomy, cigarette smoking, and age at first sexual intercourse as risk factors for prostate cancer in middle-aged men.

**DOI:** 10.1038/bjc.1988.74

**Published:** 1988-03

**Authors:** G. D. Honda, L. Bernstein, R. K. Ross, S. Greenland, V. Gerkins, B. E. Henderson

**Affiliations:** Tulane University, School of Medicine, New Orleans, Louisiana.

## Abstract

A population-based case-control study was conducted in men aged 60 or less to assess the risk of prostate cancer associated with vasectomy and other factors. Data were obtained from 216 case-control pairs by telephone interviews; this number represented 55% of all eligible cases. The matched pairs relative risk (RR) for vasectomy in ever married men was 1.4 with a 95% confidence interval (CI) of 0.9-2.3. There was a positive association between the number of years since vasectomy and prostate cancer risk (1-sided P = 0.01). Early age at first sexual intercourse was associated with increased prostate cancer risk (age less than 17 vs. 21+, RR = 2.3, 95% CI = 1.3, 4.0) but there were no consistent associations with number of sexual partners or frequency of sexual intercourse. Cigarette smoking was also associated with increased risk of prostate cancer (RR = 1.9, 95% CI = 1.2, 3.0) and there was a positive dose-response relationship with years of smoking (1-sided P = 0.001). We discuss the possible implication of the low response rate on each of these findings. To determine whether the association with vasectomy might have a hormonal basis, we compared levels of testosterone (T) and testosterone binding globulin-binding capacity (TeBG-bc) in 33 of the vasectomized control men with levels in 33 non-vasectomized controls of the same age, weight and height. T levels were higher in vasectomized than in non-vasectomized controls (1-sided P = 0.06). The ratio of T to TeBG-bc (an index of bioavailable T) was 13.5% higher in vasectomized men (1-sided P = 0.03).


*Br. J. Cancer* (1988), **57**, 326–331 © The Macmillan Press Ltd., 1988

### Vasectomy, cigarette smoking, and age at first sexual intercourse as risk factors for prostate cancer in middle-aged men

G.D. Honda[1], L. Bernstein[2], R.K. Ross[2], S. Greenland[3], V. Gerkins[2] & B.E. Henderson[2]

[1]*Tulane University, School of Medicine, New Orleans, Louisiana;* [2]*University of Southern California School of Medicine, Los Angeles California;* [3] *University of California, Los Angeles, School of Public Health, Los Angeles, California, USA.*

**Summary** A population-based case-control study was conducted in men aged 60 or less to assess the risk of prostate cancer associated with vasectomy and other factors. Data were obtained from 216 case-control pairs by telephone interviews; this number represented 55% of all eligible cases.

The matched pairs relative risk (RR) for vasectomy in ever married men was 1.4 with a 95% confidence interval (CI) of 0.9–2.3. There was a positive association between the number of years since vasectomy and prostate cancer risk (1-sided $P=0.01$). Early age at first sexual intercourse was associated with increased prostate cancer risk (age <17 *vs.* 21+, RR=2.3, 95% CI=1.3, 4.0) but there were no consistent associations with number of sexual partners or frequency of sexual intercourse. Cigarette smoking was also associated with increased risk of prostate cancer (RR=1.9, 95% CI=1.2, 3.0) and there was a positive dose-response relationship with years of smoking (1-sided $P=0.001$). We discuss the possible implication of the low response rate on each of these findings.

To determine whether the association with vasectomy might have a hormonal basis, we compared levels of testosterone (T) and testosterone binding globulin-binding capacity (TeBG-bc) in 33 of the vasectomized control men with levels in 33 non-vasectomized controls of the same age, weight and height. T levels were higher in vasectomized than in non-vasectomized controls (1-sided $P=0.06$). The ratio of T to TeBG-bc (an index of bioavailable T) was 13.5% higher in vasectomized men (1-sided $P=0.03$).

It has been hypothesized that a vasectomy may lower the risk of prostate cancer (Sheth *et al.*, 1982). This hypothesis is based in part on the observation that vasectomized rats have decreased prostate function and size (Kinson & Layberry, 1975; Pierrepoint & Davies, 1973). There is also evidence of decreased prostatic activity following vasectomy in man (Naik *et al.*, 1980). On the other hand, there are studies which indicate that vasectomy may result in increased serum testosterone levels (Johnsonbaugh *et al.*, 1975; Purvis *et al.*, 1976; Smith *et al.*, 1979), which have been hypothesized to increase prostate cancer risk (Ross *et al.*, 1983). The epidemiologic data are equivocal (Ross *et al.*, 1983; Mandel, 1981).

We conducted a prostate cancer case-control study in Los Angeles County to further test this hypothesis. Data were ascertained on each subject's surgical history, use of cigarettes and alcohol, and marital, fertility and sexual history. In order to provide further information on the association of vasectomy and circulating testosterone levels, we compared testosterone (T) and testosterone binding globulin-binding capacity (TeBG-bc) in 33 of the control men, vasectomized from 3 to 36 years earlier, and in 33 other controls of the same age, race, weight and height, who had not had a vasectomy.

### Patients and methods

Cases were men with histologically diagnosed adenocarcinoma of the prostate identified by the Cancer Surveillance Program (CSP), a population-based tumour registry covering the more than 8 million residents of Los Angeles County, California. Operational details of the registry have been previously described (Mack, 1977). Cases were retrospectively identified from January, 1979 to February, 1982, inclusively. Cases were restricted to white non-Spanish-surnamed males with no previous cancer history. Since vasectomies first became popular as a method of birth control among middle-aged men during the 1960s, the expected vasectomy prevalence among men over age 60 is low. Thus, the case group was restricted to men aged 60 or less. Blacks were not included in the case group because male sterilization is less common among US blacks than US whites (Bachrach & Mosher, 1984).

A total of 402 eligible cases were identified by the CSP and 221 of these (55%) were interviewed by telephone using a highly structured format with explicit probes. Interviews were not obtained with cases for the following reasons: physician refused permission to contact the case ($n=50$), case refused to participate ($n=22$), case died ($n=76$), case could not be located ($n=24$), or hospital did not permit case to be contacted ($n=9$).

For each interviewed case, a neighbourhood control was sought who was white, non-Spanish surnamed, with no previous cancer diagnosis, and whose date of birth was within five years of the case's date of birth. Each neighbourhood control was systematically located by contacting residential units along a pre-determined walking pattern near the case's residence at the time of diagnosis. The matched control was the first eligible control in the walking pattern who agreed to be interviewed. For 76% of the matches, this was the first eligible man identified; for 19% of the matches the second eligible man was interviewed. Overall 71 potential controls refused to participate for a control refusal rate (refusals/(refusals+interviews)) of 25%. The median number of living units contacted prior to that of the interviewed control was 11. The exposure history of controls was presumed to end at the diagnosis date of the matched case.

All interviews were conducted by one male interviewer (GH). During the interview, subjects were asked whether they had been vasectomized. Of the 45 control men who reported having had a vasectomy, 33 who had no health problems which might affect the results of this study, agreed to participate in the serological study. To minimize the potential confounding effects of age and obesity on T and TeBG-bc levels, we individually matched 33 healthy non-vasectomized males from the control pool to the eligible vesectomized males by year of birth (within 5 years), weight (within 4.5 kg), and height (within 5 cm). In addition, the vasectomized and non-vasectomized control subjects were

Correspondence: R.K. Ross, USC School of Medicine, Norris Cancer Hospital & Institute, P.O. Box 33804, 1441 Eastlake Avenue Room 803, Los Angeles, California 90033–0804, USA.
Received 30 October 1987; and in revised form, 20 January 1988.

matched within one social class grouping (based on the average education and income levels of the census tracts in which they resided) (Henderson *et al.*, 1975).

Fifteen millilitre samples of blood were drawn by venipuncture between 8 a.m. and noon in the subjects' homes by VG. The serum was stored at −20°C for time periods ranging from 1 to 4 months. Aliquots were delivered on dry ice to Endocrine Sciences Laboratories, Tarzana, CA, for determination of T (Furuyama *et al.*, 1970) and TeBG-bc levels (Nankin *et al.*, 1975). The identity of the specimens was not known to the processing laboratory. The only identifier was a coded number unique for each submission of a specimen.

We maintained the matched pairs design in the statistical analysis of questionnaire data. For dichotomous variables we used McNemar's chi-quare test and computed point estimates and exact 95% confidence limits (Breslow & Day, 1980). For continuous variables and those with multiple levels, we used conditional logistic regression methods to estimate the relative risk (RR) (Breslow & Day, 1980). For ordinal variables, a trend test was used to determine whether there was a dose-related increase or decrease in risk in the matched data.

Statistical analyses for the serological data were performed using paired *t*-tests, and repeated measures analyses of covariance to assess the significance of differences in means (Sokal & Rohlf, 1981). In addition to T and TeBG-bc, the ratio T/TeBG-bc was evaluated. This ratio is useful as an index of free T as it provides a theoretical value of T at a fixed TeBG-bc level (1.0 mcg of DHT bound $dl^{-1}$) (Udry *et al.*, 1985). Quetelet's index ($1000 \times \text{weight/height}^2$) was used as a measure of obesity. T, TeBG-bc and T/TeBG-bc followed lognormal (base 10) distributions and logarithmic (base 10) values of these variables were used in all statistical analyses. Geometric mean values are presented for serologic data. The analyses of covariance assumed a linear relationship between the covariate(s) and log hormone measures.

### Results

### *Questionnaire data*

Data on 216 case-control pairs were used in the analysis. Five unmatched interviewed cases were omitted. Forty-six percent of the controls were matched within two years of the case's date of birth and 80% were matched within four years. The age distribution for cases used in the analysis was as follows: <age 53, $n=32$; age 53–57, $n=67$; and age 58–60, $n=117$.

Neither marital status nor age at first marriage were associated with prostate cancer occurrence. Five percent of both cases and controls had never been married. Vasectomized men had slightly higher risk of prostate cancer. Thirty-five percent of the married cases and 23% of the married controls had been vasectomized (RR = 1.4, CI = 0.9–2.3). There was a positive relationship between prostate cancer risk and the number of years since vasectomy (trend test, 1-sided $P=0.01$). Compared to non-vasectomized men, men who had been vasectomized 30 or more years earlier had a RR of 4.4 for prostate cancer (Table I).

Early age at first sexual intercourse also was associated with an increased prostate cancer risk. The relative risk (age <17 *vs.* 21+) was 2.3 (CI = 1.3–4.0). Number of sexual partners was not consistently associated with risk of prostate cancer (Table II). Having sexual intercourse either infrequently (<1/week) or frequently (4+/week) three years prior to diagnosis was associated with increased prostate cancer risk when compared to intermediate levels of frequency. To determine whether the high risk associated with infrequent sexual intercourse (<1 week) was due to more advanced disease in these cases, we evaluated risk by disease stage. This bimodal effect was apparent across all disease stage strata. Men with a venereal disease history were at increased risk, although the prevalence among the controls was low (gonorrhoea 11.6% and syphilis 0.5%) and the results were statistically imprecise (Table II).

Cigarette smoking was associated with moderately increased prostate cancer risk. The RR (ever *vs.* never smoked) was 1.9 (CI = 1.2–3.0). There was a positive relationship between prostate cancer risk and smoking duration (trend test, 1-sided $P=0.001$). Men who had smoked at least 40 years had 2.6 times the risk of men who had never smoked (Table III).

Men who reported previous prostate problems had strongly increased prostate cancer risk. Most of these prostate conditions were reported to have occurred at least 10 years prior to the date of prostate cancer diagnosis. The prevalence of these problems among the cases and the associated relative risks were as follows: prostatitis (17%,

**Table I** Matched relative risks (RR) for marital status, age at first marriage, and years from vasectomy

| *Variable* | *No. cases*[a] | *No. controls*[a] | *RR* | *95% CI* | *P*[b] |
|---|---|---|---|---|---|
| Marital status | | | | | |
| Ever married | 205 | 206 | 1.0*[c] | | |
| Never married | 11 | 10 | 1.1 | 0.4, 3.3 | |
| Age at first marriage | | | | | |
| <23 | 83 | 70 | 1.0*[c] | | |
| 23–24 | 33 | 36 | 0.8 | 0.4, 1.4 | |
| 25–27 | 40 | 45 | 0.7 | 0.4, 1.3 | |
| 28+ | 40 | 45 | 0.7 | 0.4, 1.3 | 0.10 |
| Years from vasectomy | | | | | |
| 0 (none) | 138 | 151 | 1.0*[c] | | |
| 1–9 | 8 | 11 | 0.7 | 0.3, 1.9 | |
| 10–19 | 18 | 19 | 1.0 | 0.5, 2.0 | |
| 20–29 | 24 | 13 | 2.2 | 1.0, 4.8 | |
| 30+ | 8 | 2 | 4.4 | 0.9, 21.0 | 0.01 |

[a]Numbers do not always total 216 due to missing values; [b]1-sided, test for trend; [c]Analyses restricted to case-control pairs in which both men had been married.
*Referent category.

**Table II** Matched relative risks (RR) for other sexual activity variables

| *Variable* | *No. cases*[a] | *No. controls*[a] | *RR* | *95% CI* | *P*[b] |
|---|---|---|---|---|---|
| Age at first intercourse | | | | | |
| <17 | 83 | 54 | 2.3 | 1.3, 4.0 | |
| 17–18 | 51 | 59 | 1.3 | 0.7, 2.3 | |
| 19–20 | 44 | 45 | 1.5 | 0.8, 2.8 | |
| 21+ | 33 | 53 | 1.0* | | 0.002 |
| Number of sexual partners | | | | | |
| <3 | 39 | 42 | 1.0* | | |
| 3–7 | 52 | 53 | 1.1 | 0.6, 1.9 | |
| 8–20 | 52 | 48 | 1.2 | 0.6, 2.1 | |
| 21+ | 43 | 43 | 1.1 | 0.6, 2.1 | 0.36 |
| Frequency of sexual intercourse/week (3 years pre-diagnosis) | | | | | |
| <1 | 48 | 22 | 3.6 | 1.8, 7.0[c] | |
| 1 | 54 | 65 | 1.3 | 0.8, 2.3 | |
| 2 | 50 | 75 | 1.0* | | |
| 3 | 36 | 32 | 1.9 | 1.0, 3.7 | |
| 4+ | 20 | 14 | 2.5 | 1.1, 5.9 | |
| Venereal disease[d] | | | | | |
| Gonorrhoea | 33 | 25 | 1.4 | 0.8, 2.6 | |
| Syphilis | 6 | 1 | 6.0 | 0.7, 276.0 | |
| Either | 35 | 25 | 1.5 | 0.8, 2.7 | |

[a]Numbers do not always add to 216 due to missing values; [b]1-sided test for trend; [c]2-sided $P=0.0002$; [d]Reference groups differ for each analysis.
*Referent category.

Table III Matched relative risks (RR) for cigarette smoking, prostate problems, and family history of prostate cancer

| Variable | No. cases[a] | No. controls[a] | RR | 95% CI | P[b] |
|---|---|---|---|---|---|
| Years of smoking | | | | | |
| 0 | 44 | 69 | 1.0* | | |
| 1–9 | 10 | 11 | 1.7 | 0.6, 4.4 | |
| 10–19 | 22 | 25 | 1.4 | 0.7, 2.6 | |
| 20–39 | 90 | 78 | 1.9 | 1.1, 3.1 | |
| 40+ | 49 | 32 | 2.6 | 1.4, 4.9 | 0.001 |
| Prostate problem | | | | | |
| Prostatitis | 39 | 18 | 2.2 | 1.2, 4.3 | |
| Enlarged prostate | 56 | 19 | 3.6 | 2.0, 7.1 | |
| Prostate 'surgery' | 14 | 7 | 2.0 | 0.8, 5.9 | |
| Prostate cancer history in: | | | | | |
| Father | 17 | 6 | 2.8 | 1.1, 8.8 | |
| Brother | 4 | 0 | | | |
| Either father or brother | 21 | 6 | 3.5 | 1.4, 10.6 | |

[a]Numbers do not always total 216 due to missing values; [b]1-sided test for trend.
*Referent category.

RR = 2.2), enlarged prostate (25%, RR = 3.6), and prostate 'surgery' (9%, RR = 2.0) (Table III). Reported prostate problems were not verified against medical records.

There was also a strong association between prostate cancer and having a father or brother with prostate cancer (RR = 3.5, CI = 1.2–10.6) (Table III). No similar association with non-prostatic cancer among fathers and brothers was observed.

The average alcohol intake (g $day^{-1}$) was calculated from the reported intake of beer, wine, and liquor (based on 10, 13, and 20 g alcohol per drink, respectively). No consistent association was observed with increasing alcohol consumption. There were also no consistent associations between prostate cancer risk and measures of height (H), weight (W) or adjusted weight (W/H, $W/H^2$, or $W/H^3$).

Adjustment of vasectomy status and cigarette smoking history for each other or for the demographic variables, marital factors, fertility factors, and sexual history factors examined in this study, using conditional logistic regression methods, did not substantially alter the associations with prostate cancer. The association of early age at first sexual intercourse (age < 17 *vs.* later) with prostate cancer risk was reduced from RR = 1.9 to RR = 1.5, after controlling simultaneously for number of years since vasectomy and cigarette smoking duration.

### *Hormone assay results*

Characteristics of vasectomized and non-vasectomized control men contributing serum samples are compared in Table IV. Non-vasectomized men were, on average, 1.4 years older than vasectomized men (2-sided $P < 0.0001$). The two groups were closely matched on W, H and Quetelet's index. There were no important differences in the distribution of vasectomized and non-vasectomized men by the time of day that blood samples were drawn. The two groups were comparable in terms of sexual activity factors. More non-vasectomized men were current smokers (33% *vs.* 21% of vasectomized men).

Results of the serum assays are presented in Table V. The adjusted geometric mean T level (adjusted for exact time of sample collection) was 6.7% higher (1-sided $P = 0.06$) and the adjusted geometric mean T/TeBG-bc ratio was 13.5% higher (1-sided $P = 0.03$) in vasectomized than in non-vasectomized men. Vasectomized men had adjusted geometric mean TeBG-bc levels that were 4.5% lower than those of the control group (1-sided $P = 0.44$). There were only modest associations between years since vasectomy and hormone levels in the vasectomy group and between age and hormone levels overall. Adjustment for smoking status did not alter these results.

Table IV Characteristics of vasectomized and non-vasectomized men. Mean ± standard deviation presented unless otherwise noted

| Variable | Study status | |
|---|---|---|
| | Vasectomy | Non-vasectomy |
| Age at sampling | 59.7 ± 4.5 | 61.1 ± 3.9 |
| Weight (kg) | 83.4 ± 8.1 | 82.7 ± 18.8 |
| Height (cm) | 179.2 ± 6.2 | 179.5 ± 2.7 |
| Quetelet's index | 2.6 ± 0.1 | 2.5 ± 0.1 |
| Years since vasectomy | 17.7 ± 8.3 | – |
| Ever cigarette smoker | | |
| Current (%) | 21 | 33 |
| Former (%) | 40 | 43 |
| Total years smoked | 27.6 ± 14.9 | 30.5 ± 16.5 |
| Time of sample collection (a.m.) | | |
| 8:00–9:59 | 55 | 64 |
| 10:00–11:59 | 45 | 36 |
| Mean | 9:47 | 9:25 |
| Sexual factors | | |
| Age at first intercourse (%) | | |
| < 17 | 21.2 | 33.3 |
| 17–18 | 33.3 | 21.2 |
| > 18 | 45.5 | 45.5 |
| Median lifetime number of sexual partners | 9 | 6 |
| Weekly frequency of sexual intercourse (in 1979) | 2.1 ± 1.3 | 1.9 ± 1.3 |

Table V Geometric mean serum levels of testosterone (T), testosterone binding globulin-binding capacity (TeBG-bc), and ratio of T to TeBG-bc in vasectomized and non-vasectomized men

| Serum level | Study status | | 1-sided P-value[a] |
|---|---|---|---|
| | Vasectomy | Non-vasectomy | |
| T (ng $dl^{-1}$) | | | |
| Unadjusted | 495.0 (2.70 ± 0.14) | 471.7 (2.67 ± 0.18) | |
| Adjusted[b] | 499.1 | 467.8 | 0.056 |
| TeBG-bc (μg $dl^{-1}$) | | | |
| Unadjusted | 1.05 (0.020 ± 0.15) | 1.11 (0.044 ± 0.13) | |
| Adjusted[b] | 1.05 | 1.10 | 0.444 |
| T/TeBG-bc | | | |
| Unadjusted | 473.2 (2.68 ± 0.13) | 426.1 (2.63 ± 0.12) | |
| Adjusted[b] | 478.5 | 421.4 | 0.029 |

[a]Based on repeated measures analysis of covariance F test, adjusting for age when blood was drawn and time of day that blood was drawn (using minutes elapsed since 8 a.m.); [b]Adjusted to a mean time of blood drawing of 96.7 min past 8 a.m. and to 59.9 years of age.

### Discussion

The major findings of this study are the moderately strong relationships between prostate cancer risk and cigarette smoking duration, first intercourse before age 17 and, for married men, interval since vasectomy. Family history of prostate cancer and past prostatic diseases are also strong predictors of risk, but in the absence of medical record validation, we cannot eliminate the possibility of recall bias for these two findings. Previous case-control studies of the relationship between benign prostatic hypertrophy and prostate cancer have produced conflicting results (Armenian *et al.*, 1974; Greenwald *et al.*, 1974). However, the familial association of prostate cancer mortality using the virtually complete records of the Church of the Latter Day Saints provides support for the familial risk detected here (Woolf, 1960).

The growth and development of the prostate is under the control of testosterone and its metabolite dihydrotestosterone, and there is considerable evidence supporting a hormonal etiology for prostate cancer (Noble, 1982; Ghanadian *et al.*, 1978; Ahluwalia *et al.*, 1981; Drafta *et al.*, 1982; Ross *et al.*, 1986). Our data on hormone levels in vasectomized and non-vasectomized men provide limited support that the observed effect of vasectomy may have a hormonal basis. While we find no major differences in either T or TeBG-bc in vasectomized compared to non-vasectomized men, when measured many years after the procedure, our data do suggest that vasectomized men have higher levels of biologically available T.

A number of prospective studies have examined the effect of vasectomy on hormonal status, but the results are equivocal; and generally, they are based on small numbers of study subjects and have examined subjects within a short time (no more than 2 years) after vasectomy. Several of these studies reported no changes in hormonal status following a vasectomy (Goebelsmann *et al.*, 1979; Alexander *et al.*, 1980; de la Torre *et al.*, 1983). Others suggested that serum T levels may increase slightly after vasectomy (Johnsonbaugh *et al.*, 1975; Purvis *et al.*, 1976). One study, with the longest follow-up time (3 years) and largest sample, reported a statistically significant increase in the mean serum T levels of vasectomized men (Smith *et al.*, 1979). Two case-control studies evaluated plasma T levels in non-vasectomized men and in men vasectomized from 1 to 5 years earlier (Varma *et al.*, 1975; Skegg *et al.*, 1976). Neither found statistically significant differences in T levels. In one of these studies, the number of non-vasectomized men used for comparison was small ($n = 16$) and may not have been adequate to demonstrate a difference (Varma *et al.*, 1975). The second study compared 188 vasectomized men with 100 men scheduled for a vasectomy and found mean T levels that were about 10% higher in the vasectomy group (Skegg *et al.*, 1976). None of these studies examined TeBG-bc levels or indices of free T.

While the small differences observed in our study could be an effect of the surgical procedure itself (Johnsonbaugh *et al.*, 1975; Purvis *et al.*, 1976; Smith *et al.*, 1979; Gupta *et al.*, 1975), other explanations seem equally plausible. One possible explanation is that the decision to have a vasectomy is closely related to sexual practices (i.e., men with high levels of sexual activity choose vasectomy as a form of birth control more frequently than sexually inactive men). In turn, heightened sexual activity may be related to increased circulating bioavailable androgens (Tsitouras *et al.*, 1982), which may be the more immediate cause of prostate cancer development.

Cigarette smoking has not been associated with increased risk of prostate cancer in most previous epidemiologic studies (Hammond, 1966; Doll & Peto, 1976; Wynder *et al.*, 1971; Armenian *et al.*, 1975; Jackson *et al.*, 1980). Although the majority of these studies were hospital-based case-control studies and used controls who were not screened for diseases potentially associated with smoking, most cohort studies which have examined this relationship also have been negative. Because of the largely negative results from previous studies, information collected on smoking habits in the present study was related to duration only. No data on number of cigarettes smoked daily were available. Nonetheless, the smoking association with prostate cancer observed here also could have a hormonal basis. Cigarette smoking has been shown to be associated with high circulating testosterone levels in middle-aged men (Dai *et al.*, 1981; Deslypere & Vermeulen, 1984) although it is unclear whether this is a cause and effect relationship. One hypothesis to explain both an association between smoking and prostate cancer that is limited to younger men and a non-causal association between smoking and testosterone levels, is that socio-cultural determinants of cigarette smoking in the US are age-related. In younger cohorts, in which the overall prevalence of smoking is low, regular use of cigarettes may be more closely linked with risk-taking tendencies and, perhaps, elevated circulating testosterone levels.

The increased prostate cancer risk associated with early age at first intercourse in this study has been observed by others (Mishina *et al.*, 1985). Others also have found evidence that additional factors related to increased sexual activity (frequency of intercourse, number of sexual partners, history of venereal disease) increase prostate cancer risk (Steele *et al.*, 1971; Krain, 1974; Heshmat *et al.*, 1975). In the present study venereal disease was associated with increased risk, but the association with frequency of intercourse was complex. Findings such as these have suggested the possibility that prostate cancer may be caused by transmission of an infectious agent through sexual activity. However, in a cohort study of cancer mortality in Catholic priests in Los Angeles we found a small, but statistically non-significant, excess of prostate cancer deaths (Ross *et al.*, 1981). The absence of a marked deficit of prostate cancer mortality among such men is evidence against sexual transmission of the disease. An alternative explanation is that early age at first intercourse is indicative of increased sexual activity or higher 'sex drive' among the cases and is another indirect measure of circulating androgen levels.

We conclude that it is possible that the observed risk factors of vasectomy, cigarette smoking, and early age at first intercourse could all be explained by a single aetiologic hypothesis related to high circulating testosterone. Further study is needed to verify these associations and, if real, their physiological basis. Further study is also needed to determine whether the higher T/TeBG-bc ratio in vasectomized men observed in our study is pre-existing (as might occur if sexual practices are correlates of both circulating T levels and of the choice of vasectomy as an elective birth control method), or whether the procedure itself results in higher bioavailable T levels.

Only 55% of eligible cases were interviewed in this study, raising important questions about the representativeness of the interviewed cases to all eligible cases in terms of the variables evaluated. The high rate of deaths (19% of all eligible cases) is of particular concern, and is related, in part, to our decision to interview retrospectively identified cases. As expected, interviewed cases tended to have less advanced disease at diagnosis, but were similar to non-interviewed cases on other demographic variables routinely collected by the CSP, including religion, occupation, median income of the census tract of residence, and mean age at diagnosis. However, these similarities offer no great reassurance that some of the factors being investigated might have influenced outcome (or other losses) rather than disease development. If anything, we might expect survivors to be underrepresented by smokers given the myriad of health problems associated with smoking. In addition, since prostate cancer growth is often testosterone dependent, if the risk factors identified in this study are indices of testosterone production as hypothesized above, we again would expect that survivors might be underrepresented by individuals 'positive' for these variables. To the extent that any of the losses in this study were social class related, our use of neighbourhood controls with matched analyses should have minimized bias.

A second methodologic concern in interpreting the results of this study is the validity of information collected by a telephone interview, particularly for variables dealing with sexual activity, contraceptive practices and other 'sensitive' areas. Vasectomies have become a common method of male sterilization and there appears to be no social stigma associated with the procedure (Uehling & Wear, 1972). Based on interviews, there is nearly 100% agreement between reported vasectomy status and medical records (Massey *et*

al., 1985), but we know of no comparable data based on telephone interviews. Only 3 questions dealing specifically with sexual activity were asked in this study and these were placed at the end of the interview, to allow good rapport to develop between the interviewer and the participant. Although every participant provided an estimate of age at first intercourse, 10% of cases and 5% of controls either refused or could not estimate their number of sexual partners. One could speculate about possible non-random misclassification of responses (e.g. cases providing more accurate responses due to personal interest in the study or controls choosing more conservative, 'safer' responses because of lingering concerns about how the information would be used), but we believe it more likely that misclassification occurred to a comparable degree among cases and controls, resulting in underestimations of any true associations.

Supported by Grant CA17054, from the National Cancer Institute, National Institutes of Health, Bethesda, MD.

### References

AHLUWALIA, B., JACKSON, M.A., JONES, G.W., WILLIAMS, A.O., RAO, M.S. & RAJGURU, S. (1981). Blood hormone profile in prostate cancer patients in high-risk and low-risk populations. *Cancer*, **48**, 2267.

ALEXANDER, N.J., FREE, M.J., PAULSON, C.A., BUSCHBOM, R. & FULGHAM, D.L. (1980). A comparison of blood chemistry, reproductive hormones, and the development of antisperm antibodies after vasectomy in men. *J. Androl.*, **1**, 40.

ARMENIAN, H.K., LILIENFELD, A.M., DIAMOND, E.L. & BROSS, I.D.J. (1975). Epidemiologic characteristics of patients with prostatic neoplasms. *Am. J. Epidemiol.*, **102**, 47.

ARMENIAN, H.K., LILIENFELD, A.M., DIAMOND, E.L. & BROSS, I.D. (1974). Relation between benign prostatic hyperplasia and cancer of the prostate. *Lancet*, **i**, 115.

BACHRACH, C.A & MOSHER, W. D. (1984). *The Use of Contraception in the United States, 1982*. NCHS Advance Data, 102, Vital and Health Statistics of the National Center for Health Statistics, Hyattsville, MD.

BRESLOW, N.E. & DAY, N.E. (1980). The analysis of case-control studies. In *Statistical Methods in Cancer Research*, Vol. I; International Agency for Research on Cancer. Lyon, France.

DAI, W.S., KULLER, L.H., LAPORTE, R.E., GUTAI, J.P., FALVO-GERARD, L. & CAGGIULA, A. (1981). The epidemiology of plasma testosterone levels in middle-aged men. *Am. J. Epidemiol.*, **114**, 804.

DE LA TORRE, B., HEDMAN, M., JENSEN, F., PEDERSON, P.H. & DICZFALUSY, E. (1983). Lack of effect of vasectomy on peripheral gonadotrophin and steroid levels. *Int. J. Androl.*, **6**, 125.

DESLYPERE, J.P & VERMEULEN, A. (1984). Leydig cell function in normal men: Effect on age, life-style, residence, diet and activity. *J. Clin. Endocrinol. Metab.*, **59**, 955.

DOLL, R. & PETO, R. (1976). Mortality in relation to smoking: 20 year's observations on male British doctors. *Br. Med. J.*, **2**, 1525.

DRAFTA, D., PROCA, E., ZAMFIR, V., SCHINDLER, A.E., NEASCU, E. & STROE, E. (1982), Plasma steroids in benign prostatic hypertrophy and carcinoma of the prostate. *J. Steroid. Biochem.*, **17**, 689.

FURUYAMA, S., MAYES, D.M. & NUGENT, C.A. (1970). A radioimmunoassay for plasma testosterone. *Steroid*, **16**, 415.

GHANADIAN, R., PUAH, C.M. & O'DONOGHUE, E.P.N. (1978). Serum testosterone and dihydrotestosterone in carcinoma of the prostate. *Br. J. Cancer*, **39**, 696.

GOEBELSMANN, U., BERNSTEIN, G.S., GALE, J.A. & 4 others (1979). Serum gonadotropin, testosterone, estradiol and estrone levels prior to and following bilateral vasectomy. In *Vasectomy: Immunologic Pathophysiologic Effects in Animals and Man*. Lepow, I.H. & Crozier, R. (eds.), p. 165, Academic Press, New York.

GREENWALD, P., KIRMS, S.V., POLAN, A.K. & DICK, V.S. (1974). Cancer of the prostate among men with benign prostatic hyperplasia. *J. Natl Cancer Inst.*, **53**, 335.

GUPTA, A.S., KOTHARI, L.K., DHRUVA, A. & BAPNA, R. (1975). Surgical sterilization by vasectomy and its effect on the structure and function of the testis in man. *Br. J. Surg.*, **62**, 59.

HAMMOND, E.C. (1966). Smoking in relation to the death rates of one million men and women. *J. Natl Cancer Inst. Monogr.*, **19**, 127.

HENDERSON, B.E., GORDON, R.J., MENCK, H., SOOHOO, J., MARTIN, S.P. & PIKE, M.C. (1975). Lung cancer and air pollution in south central Los Angeles County. *Am. J. Epidemiol.*, **101**, 477.

HESHMAT, M.Y., KOVI, J., HERSON, J., JONES, G.W. & JACKSON, M.A. (1975). Epidemiologic association between gonorrhea and prostatic carcinoma. *Urology*, **6**, 457.

JACKSON, M.A., KOVI, J., HESHMAT, M.Y. & 8 others (1980). Characterization of prostatic carcinoma among blacks: A comparison between a low-incidence area, Ibadan, Nigeria and a high-incidence area, Washington, D.C.. *Prostate*, **1**, 185.

JOHNSONBAUGH, R.E., O'CONNEL, K., ENGEL, S.B., EDSON, M. & SODE, J. (1975). Plasma testosterone, luteinizing hormone and follicle stimulating hormone after vasectomy. *Fert. Steril*, **26**, 329.

KINSON, G.A. & LAYBERRY, R.A. (1975). Long-term endocrine responses to vasectomy in the adult rat. *Contraception*, **11**, 143.

KRAIN, L.S. (1974). Some epidemiologic variables in prostate carcinoma in California. *Prev. Med.*, **3**, 154.

MACK, T.M. (1977). Cancer Surveillance Program in Los Angeles County. *J. Natl Cancer Inst. Monogr.*, **47**, 99.

MANDEL, J.S. (1981). Epidemiologic study of etiologic factors in prostate cancer. University of Minnesota, 1981 Unpublished (dissertation).

MASSEY, F.J., BERNSTEIN, G.S., O'FALLON, W.M. & 17 others (1985). Vasectomy and health: Results from a large cohort study. *J. Am. Med. Assoc.*, **52**, 1023.

MISHINA, T., WATANABE, H., ARAKI, H. & NAKAO, M. (1985). Epidemiological study of prostatic cancer by matched-pairs analysis. *Prostate*, **6**, 423.

NAIK, V.K., JOCHI, U.M. & SHETH, A.R. (1980). Long-term effects of vasectomy on prostatic function in men. *J. Reprod. Fert.*, **58**, 289.

NANKIN, H.R., PINTO, R., FAN, D.F. & TROEN, P. (1975). Daytime titers of testosterone, LH, estrone, estradiol and testosterone-binding protein: Acute-effects of LH and LH-releasing hormone in men. *J. Clin. Endocrinol. Metab.*, **41**, 271.

NOBLE, R.L. (1982). Prostate carcinoma of the Nb rat in relation to hormones. *Int. Rev. Exp. Pathol.*, **23**, 113.

PIERREPOINT, C.G. & DAVIES, P. (1973). The effects of vasectomy on the activity of prostatic RNA polymerase in rats. *J. Reprod. Fert.*, **35**, 149.

PURVIS, K., SAKSENA, S.K., CEKAN, Z., DICZFALUSY, E. & GINER, J. (1976). Endocrine effects of vasectomy. *Clin. Endocrinol.*, **5**, 263.

ROSS, R.K., BERNSTEIN, L., JUDD, H., HANISCH, R., PIKE, M. & HENDERSON, B.E. (1986). Serum testosterone levels in healthy young black and white men. *J. Natl Cancer Inst.*, **76**, 45.

ROSS, R.K., DEAPEN, D., CASAGRANDE, J., PAGANINI-HILL, A. & HENDERSON, B.E. (1981). A cohort study of mortality from cancer of the prostate in Catholic priests. *Br. J. Cancer*, **43**, 233.

ROSS, R.K., PAGANINI-HILL, A. & HENDERSON, B.E. (1983). Etiology of prostate cancer: What does the epidemiology suggest? *Prostate*, **4**, 333.

SHETH, A.R. & PANSE, G.T. (1982). Can vasectomy reduce the incidence of prostate tumor? *Medical Hypothesis*, **8**, 237.

SKEGG, D.C.G., MATTHEWS, J.D., GUILLEBAUD, J. & 6 others (1976). Hormonal assessment before and after vasectomy. *Br. Med. J.*, **1**, 621.

SMITH, K.D., TCHOLAKIAN, R.K., CHOWDHURY, M. & HSI, B.P. (1979). Endocrine studies in vasectomized men. In *Vasectomy: Immunologic and Patho-physiologic Effects in Animals and Men* Lepow, I.H. & Crozier, R. (eds), Academic Press, New York.

SOKAL, R.R. & ROHLF, F.J. (1981). *Biometry: The Principles and Practice of Statistics in Biological Research*, 2nd Edition. W.H. Freeman and Company, San Francisco.

STEELE, R., LESS, R.E.M., KRAUS, A.S. & RAO, R. (1971). Sexual factors in the epidemiology of cancer of the prostate. *J. Chron. Dis.*, **24**, 29.

TSITOURAS, P.D., MARTIN, C.E. & HARMAN, S.M. (1982). Relationship of serum testosterone to sexual activity in healthy elderly men. *J. Geronotol.*, **37**, 288.

UDRY, J.R., BILLY, J.O., MORRIS, N.M., GROFF, T.R. & RAJ, M.H. (1985). Serum androgenic hormones motivate sexual behavior in adolescent boys. *Fertil. Steril*, **43**, 90.

UEHLING, D.T. & WEAR, J.B. (1972). Patients attitudes towards vasectomy. *Fert. Steril*, **23**, 838.

VARMA, M.M., VARMA, R.R., JOHANSON, A.J., KOWARSKI, A. & MIGEON, C.J. (1975). Long-term effects of vasectomy on pituitary-gondal function in man. *J. Clin. Endocrinol Metab.*, **40**, 868.

WOOLF, C.M. (1960). An investigation of the familial aspects of cancer of the prostate. *Cancer*, **13**, 739.

WYNDER, E.L., MABUCHI, K. & WHITMORE, W.F. (1971). Epidemiology of cancer of the prostate. *Cancer*, **28**, 344.